# Treatment of Boiler Condensate by Ultrafiltration for Reuse

**DOI:** 10.3390/membranes12121285

**Published:** 2022-12-19

**Authors:** Grégory Cano, Philippe Moulin

**Affiliations:** Laboratoire de Mécanique, Aix-Marseille University, Modélisation et Procédés Propres, Equipe Procédés Membranaire (EPM-M2P2-CNRS-UMR 7340), Europôle de l’Arbois, BP 80, Bat. Laennec, Hall C, CEDEX 04, 13545 Aix en-Provence, France

**Keywords:** ultrafiltration, condensate, reuse, boiler, hydrocarbons

## Abstract

The generation of water vapor is crucial for the petrochemical industry. In order to protect the boiler from damage, the re-injected water must not contain any suspended matter, especially hydrocarbons. Moreover, it is condensed steam with a temperature close to 100 °C and the unintentional creation or chronic generation of pollution, respectively, that can more or less produce the concentrated pollution. In this context, membrane processes appear promising in order to achieve this reuse and more especially crossflow ceramic membranes. The novelty of this paper is to study the retention of hydrocarbons and suspended solids contained in the condensate hot water of a high-capacity boiler using ceramic ultrafiltration membranes. In total, two ultrafiltration molecular weight cut-offs were used: 50–150 kDa. Several operating parameters were studied such as effluent type (accidental or chronic pollution), temperature, transmembrane pressure, initial volume, and pilot plant size. In all cases, retention of suspended matter was above 90% and residual hydrocarbon concentrations were under 0.1 ppm even for high-volume concentrations. Control of the transmembrane pressure and the molecular weight cut-off of the membrane are key to optimizing the process. Despite the high-volume concentration obtained, the membranes were perfectly regenerated with conventional cleaning procedures.

## 1. Introduction

In the petrochemical industry, the use of high-capacity boilers is imperative to execute the various processes involved. This study concerns the treatment of condensate-containing hydrocarbon pollution, in order to reuse the condensate in a large-capacity boiler. The steam produced by the boiler thus passes through heat exchangers, reheating lines, turbines, etc. The hot condensed part is collected as condensate and then reinjected into the boilers that must be free of hydrocarbons (HC) and particles (<5 ppm) and have a low turbidity to avoid possible damage. The oil present in wastewater can be found as free or dispersed with large droplets, emulsified, and dissolved. Different treatments such as chemical and biological processes have been developed to treat this type of effluent. However, these processes do not work at high temperatures. Moreover, they must not be expensive, use toxic chemicals, need space for installation or produce secondary pollution [1]. For years, membrane technology has shown its reliability for the separation of oil wastewater [2,3,4,5]. In the context of the petroleum site studied, the current filter treatment is insufficient for chronic pollution in terms of hydrocarbon retention but becomes irreversibly fouled after a few hours during accidental pollution. Ceramic is resistant to extreme environmental conditions, especially temperature [6]. Ceramic membranes are widely used in industry for this kind of application due to their thermal stability and ability to handle strong solvents [7,8]. In addition, consistent with the hydrocarbon limit, such limits have been achieved in permeate with similar hydrocarbon values in the effluent and concentration factors of up to 60 [9]. In this way, the ultrafiltration membrane process is considered effective for purification [4] even if permeate flux decreases over time due to membrane fouling [10]. This inevitably leads to chemical cleaning of the membrane where the effectiveness of using alkaline and acidic cleaning agents at high temperatures for oil wastewater has been widely studied [11,12]. Parameters such as transmembrane pressure (TMP) or velocity of the effluent in the vicinity of the membrane must be optimized to reduce the fouling phenomenon and thus the frequency of chemical cleaning [13,14,15,16,17]. The innovative aspect of this study is to consider, for the first time, a membrane process, and more precisely ultrafiltration, to realize the reuse of boiler condensate in real conditions up to tests on an industrial site. The objectives of this study are therefore to (i) treat chronic pollution which may fluctuate in hydrocarbon concentration between 2 and 5 ppm and (ii) treat accidental pollution, which is much higher ranging between 50 and 100 ppm over a very short period. An ultrafiltration process was used for the treatment of this effluent at very high temperatures (90–100 °C) and high flow rate. This study aimed to define a membrane process to treat the 300 m^3^.h^−1^ of condensate produced by the industrial site. For this, a crossflow filtration in a closed-loop system was used to concentrate the pollution and thus evaluate the membrane efficiency to retain a high-pollutant load.

## 2. Materials and Methods

### 2.1. Effluent

As the first tests were carried out at the laboratory scale, two types of condensates were provided by the petrochemical plant (Pétroineos, Fos sur Mer, France) corresponding to (a) chronic pollution with a very low concentration of hydrocarbon ([HC] < 5 ppm) and (b) a higher concentration of accidental pollution ([HC] > 50 ppm). This HC concentration was not easy to determine during the analysis due to the large degree of heterogeneity of the sample; however, a value greater than 50 ppm was obtained. Indeed, the presence of a large quantity of oil emulsion supernatant was observed in the effluent. At the laboratory scale, two samples of gasoline AO09 and fuel CG09 were added (100 ppm) to the industrial condensate to create mixed effluents to simulate accidental pollution. This kind of mixing turned out to be complicated to achieve and required an ultrasonic bath at high temperature to obtain a representative homogeneous solution. Finally, the last experiments were carried out at a semi-industrial scale, directly at the petrochemical plant, where the effluent to be treated was collected after the pre-filter and before the inlet of the boiler. A summary of the characteristics of the effluent is presented in Table 1.

### 2.2. Membranes

Ceramic membranes were used in this study, provided by the CTI manufacturer (ALSYS group) from their KLEANSEP product line. These are tubular-shaped filters with an asymmetrical membrane structure that have the advantages of high mechanical resistance materials, with the ability to withstand high temperatures (>150 °C) and high levels of oils and solids, together with a compatibility with the most harsh chemicals. Thus, a 150 kDa Molecular Weight Cut-Off (MWCO) (19 channels) and two 50 kDa (7 and 52 channels) were used. Their characteristics are presented in Table 2.

### 2.3. Apparatus

**Figure 1:**
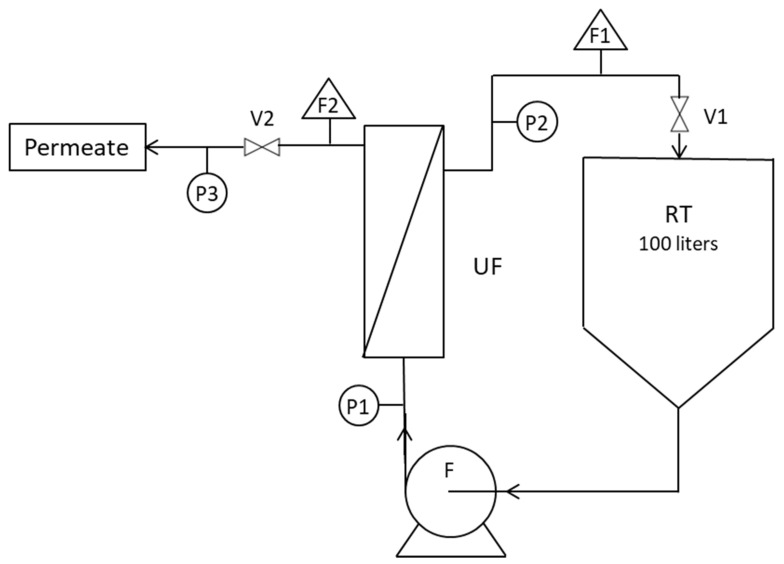
Laboratory pilot plant; UF—ultrafiltration module; RT—retention tank; F—feed pump; P1/2/3—pressure sensors; F1/2—flow meters; V1/2—valves.

**Figure 2:**
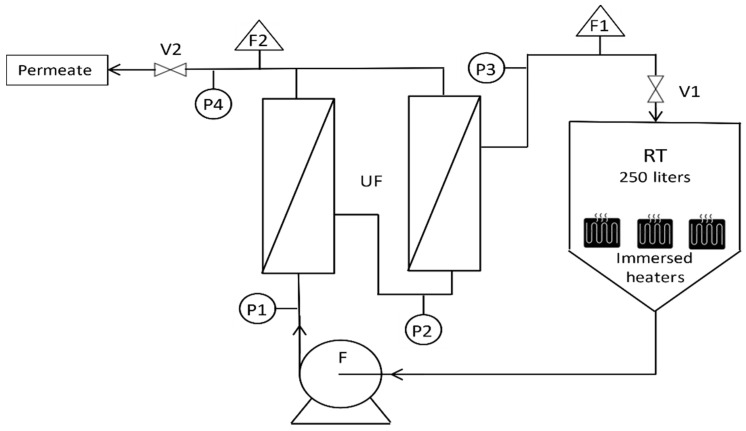
Semi industrial pilot plant; UF—ultrafiltration modules; RT—retention tank with immersed heaters; F—feed pump; P1/2/3/4—pressure sensors; F1/2—flow meters; V1/2—valves.

The concentration of hydrocarbons was processed by the SEM (Société des Eaux de Marseille, France) company using the NF T 90-114 method which is based on the determination of total hydrocarbons by infrared spectrophotometry with a detection limit <0.1 mg L^−1^. Turbidity was measured with a Turb 550 IR turbidimeter from WTW (Berlin, Germany) with a 2% relative error, electrical conductivity with a (Sension + EC7) conductimeter from Hach (Berlin, Germany) and pH with a (Sension + pH31) pH-meter from Hach (USA). The experimental set-up used for the filtration tests at ambient temperature was of a basic design allowing feed volumes of 100 L to be treated using a single membrane (Figure 1). Pressure sensors were placed upstream and downstream of the membrane and at the permeate outlet with a control valve to regulate the transmembrane pressure (TMP). For high-temperature experiments, the semi-industrial plant used membranes that were also provided by the CTI manufacturer, these allowing larger volumes to be processed, and the use of two membranes simultaneously in series (Figure 2). In addition, the process was equipped with 3 heating resistances allowing high temperatures to be reached. Cross-flow velocity was fixed at 3 m s^−1^ in the membrane channels for all experiments to prevent them from fouling, especially when high VCF values were reached. Permeate flux (L h^−1^ m^−2^) was determined by measuring the mass of permeate collected over time and then brought back to the reference temperature of 20 °C using the dynamic viscosity of ultrapure water [18]. These permeability results, Lp, were calculated using the transmembrane pressure TMP (bar). This semi-industrial plant is used for high-temperature tests in the laboratory with sample condensates and on the petroleum site for on-line tests.

## 3. Results

### 3.1. Filtration at Laboratory Scale

**Figure 3:**
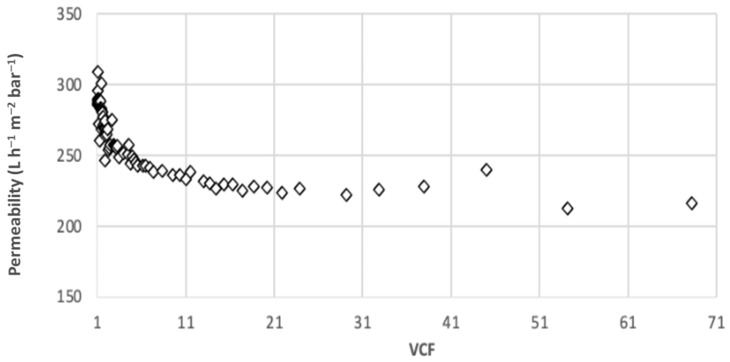
Permeability as a function of VCF [MWCO 150 kDa, PTM = 1 bar, T = 20°C].

The first experiments were carried out with 300 L of chronic effluent at laboratory scale, for a constant TMP of 1 bar and with a MWCO of 150 kDa. Figure 3 presents the permeability as a function of the volume concentration factor (VCF). The permeability decreases from 310 to 230 L h^−1^ m^−2^ bar^−1^ for a VCF < 15, then tend to stabilize at 230 L h^−1^ m^−2^ bar^−1^ to reach a VCF value of 70. Table 3 shows the results of analysis regarding pH, conductivity, turbidity, and [HC]. For a VCF of 70, analyses of the final retentate indicate a [HC] of 15 mg L^−1^ with a turbidity of 26 NTU, and for the permeate a [HC] of 0.2 mg L^−1^ with a turbidity less than 0.4 NTU which comply with the boiler standards. As expected for the ultrafiltration membrane, no real pH and conductivity variations were observed. The suspended matter retention varied from 68 to 98.8%. For HC, a good agreement was obtained between the final value and the initial value and VCF: a low quantity of HC is fixed on the membrane. In the end, a HC retention of 99% was obtained. The quality of the permeate was sufficient to be reused in the boiler. Furthermore, 100% of the membrane permeability was recovered with conventional chemical cleaning (acid/base).

**Figure 4:**
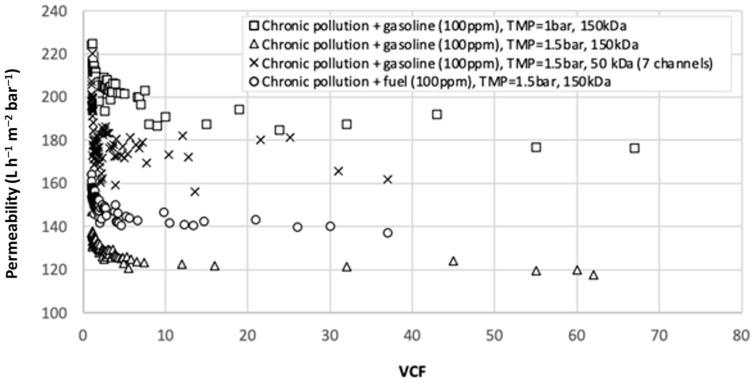
Permeability as a function of VCF [MWCO 150/50 kDa, PTM = 1/1.5 bar, T = 20°C].

The subsequent experiments were intended to achieve a higher initial concentration of hydrocarbons simulating an accidental pollution. For that purpose, two samples of gasoline and one of fuel were mixed with the chronic [HC] effluent. Figure 4 presents the permeability as a function of VCF for four experiments: (1) 150 kDa—chronic effluent + 100 ppm of gasoline at TMP = 1 bar, (2) 150 kDa—chronic effluent + 100 ppm of gasoline at TMP = 1.5 bar, (3) 150 kDa—chronic effluent + 100 ppm of fuel at TMP = 1.5 bar and (4) 50 kDa—chronic effluent + 100 ppm of gasoline at TMP = 1.5 bar. Table 4 shows the analysis results regarding pH, conductivity, turbidity, and [HC] for initial and final VCF. The permeability decreased nearly identically in all cases regardless of the variation in TMP and irrespective of the type of pollutant used. Results for the mixed solution with gasoline showed us that permeability of the membrane 150 kDa decreased by 35% by increasing the TMP from 1 bar to 1.5 bar. This can be explained by a faster and significant fouling of the membrane in the first moments of filtration when the TMP is high. Thus, TMP is a very sensitive parameter that must be adjusted correctly to achieve optimal permeabilities. Whatever the solution, the [HC] in the permeate was very low (<0.4 ppm) for the first permeate and undetectable (<0.1 ppm) even for a high VCF. A high retention of suspended matter was observed with turbidity in the permeate lower than 1 NTU and retention above 98% for the final VCF. Concerning the evolution of the permeability at low MWCO (50 kDa), we observed an initial decrease to a VCF value of 1.5 and a stabilization around 170 L h^−1^ m^−2^ bar^−1^ until the final VCF value of 37 was reached. The effect of MWCO and TMP on performance led to the same conclusion: a high TMP or MWCO seemed to cause a faster or higher permeability drop, which could be explained by stronger internal fouling. Higher TMP resulted in droplets passing rapidly through the membrane pores, so more oil droplets accumulate on the membrane surface and consequently in the membrane pores, leading to membrane fouling [5]. The authors observed that permeate flux no longer increases beyond a value of 1.25 bar resulting in a drop in total organic carbon removal efficiency. Thus, an MWCO of 50 kDa and a TMP of 1 bar appear to be the best conditions for further experiments at high temperatures on the petroleum site. Even for accidental pollution, the quality of the permeate was sufficient to be reused in the boiler. In fact, the [HC] for the final permeate was under 0.1 mg L^−1^ in all cases which is the detection limit, as well as for the turbidity.

### 3.2. Filtration with Semi-Industrial Pilot Plant at High Temperature

**Figure 5:**
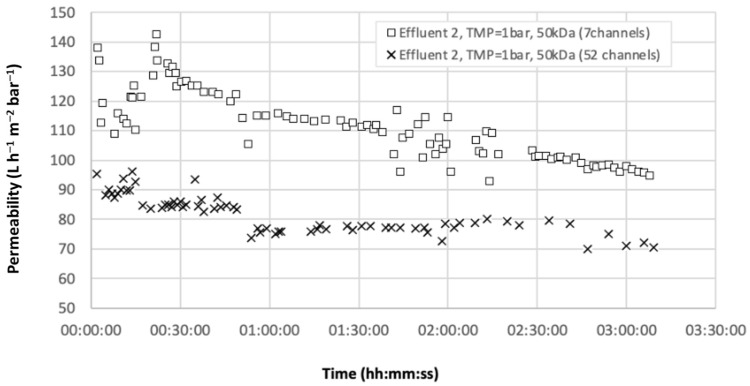
Permeability as a function of time [MWCO 50 kDa, 7/52 channels, PTM = 1 bar, T = 70 °C].

Experiments were then carried out with semi-industrial set-up at high temperature with the accidental effluent. A temperature of 65 °C was reached with the use of immersion heaters due to the external temperature (20 °C). The initial volume of the feed tank was set to 250 L. Unlike the pilot used in the laboratory, which had a funnel-shaped bottom, this one had a flat bottom which caused a drop in flow when the remaining volume of the feed tank was less than 25 L and thus prevented reaching high VCF. Figure 5 presents the evolution of permeability (at 20 °C) as a function of time for the two different 50 kDa membranes (7 and 52 channels), and Table 5 provides the analysis results. Thus, at the very beginning of filtration, permeability was around 90 L h m^2^ bar^−1^ for the 52 channels and 115 L h^−1^ m^−2^ bar^−1^ for the 7 channels which measured 25% higher. Although the permeability decreased linearly for the 7 channels to reach 95 L h^−1^ m^−2^ bar^−1^ at the end, the 52 channels tended to stabilize at just under 80 L h m^2^ bar^−1^ between VCF values of 1.5 and 5. The permeability drop after this value (last points) was due to the flat bottom problem of the tank mentioned above. In all cases, turbidity retention was above 90% at the final VCF and [HC] were under 0.1 ppm. The difference in permeability could also be explained by a lower Reynolds number in the 52 channel membrane (around 12,000) than in the 7 channel membrane (around 33,000) for an identical flow rate. Filtration at ambient temperature or higher (65 °C—limited by the ambient temperature of the laboratory) did not modify the retention of HC. The turbidity of the permeate was also very low, allowing the reuse of boiler condensates after ultrafiltration.

### 3.3. Filtration with Semi-Industrial Pilot Plant on Line

**Figure 6:**
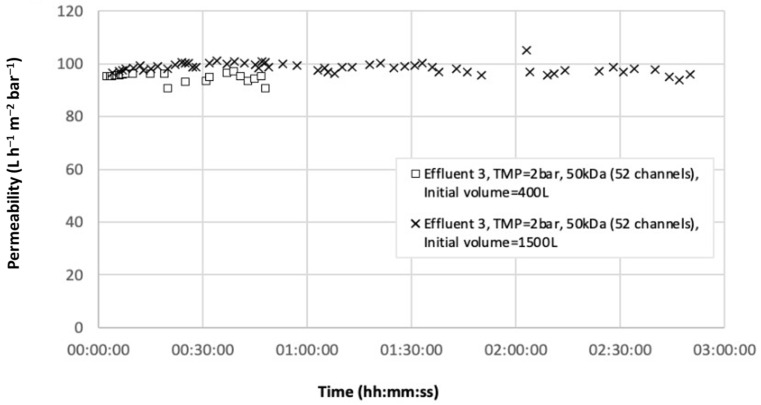
Permeability as a function of time.

The last experiments were carried out at the petrochemical plant using two 50 kDa membranes (52 channels) in series. The semi-industrial pilot was positioned near the boiler where the hot condensate was collected from a tap on the pipe. Temperatures at this location were around 70 degrees and were kept constant. This real temperature was close to the hot temperature tested in the laboratory. This configuration enabled the container to be filled throughout the experiment, allowing the use of effluent volumes greater than the initial capacity. Figure 6 presents the permeability as a function of time for initial volumes of 400 and 1500 L, and Table 6 show the results of analyses. In both cases, permeability remained stable at values between 90 and 100 L h^−1^ m^−2^ bar^−1^, reaching VCF values of 36 for the 400 L initial volume and 50 for the 1500 L. As mentioned before, the flat bottom of the retention tank prevented higher values to be reached. Retention of suspended matter was about 45.6% at the beginning due to the low turbidity of the effluent, but turbidity was lower than 1 NTU in the permeate. At the end of the process, suspended matter retention was above 92%. The [HC] was undetectable (<0.1 ppm). These two conditions enabled the reuse of boiler condensates after ultrafiltration. These results were consistent with other studies. Bilstad and Espedal [19] used UF organic tubular membranes with MWCO between 100 and 200 kDa to treat effluent from oilfield-produced water but at a low temperature (60 °C). Results showed that permeability varied from 23 to 55 L h^−1^ m^−2^ bar^−1^ with TMP between 6 and 10 bars and the total hydrocarbon concentration could be reduced to 2 mg L^−1^ from 50 mg L^−1^ (96% removal). The high concentration of HC in the final permeate likely resulted from the high TMP applied, which reduced the permeability and decreased the removal efficiency (as mentioned above), and/or the choice of the membrane materials. Silvio et al. [20] estimated a permeability of between 195 and 380 L h^−1^ m^−2^ bar^−1^ using ceramic UF membranes with a pore size of 0.1 µm and a TMP of 1.5 bar at 45 °C from an oil production offshore unit. The concentration of oil and grease decreased from 25 to less than 3 mg L^−1^ (88% removal). Permeabilities obtained were very high and the authors claimed that this could be attributed to the higher salt content in the effluent. High-ionic concentration diminishes the double-layer thickness around the emulsion droplets, reducing the electrostatic barrier to coalescence, promoting larger oil droplets and consequently, lower permeation resistance [21].

## 4. Conclusions

For the first time, an ultrafiltration process was used to realize the reuse of boiler condensate in real conditions up to tests on an industrial site. Experiments were first performed at room temperature under a laboratory setting and then at high temperature to select the MCWO that would allow reuse of the permeate in the boiler and to determine whether temperature impacted the retention of HC. Secondly, on-site tests were performed to validate these conclusions. Laboratory tests showed significant VCF values with a permeability of around 200 and 170 L h^−1^ m^−2^ bar^−1^ for molecular cut-off values of 150 kDa and 50 kDa, respectively. The lower MWCO values meet expectations in terms of hydrocarbon retention. For the operating conditions that were used (temperature, concentration, TMP, etc.), the results indicated good retention of the suspended matter in all cases (>90%) with a hydrocarbon concentration under 0.1 ppm in the final permeate, except for the 150 kDa at high VCF (70) for chronic pollution at room temperature: the choice of a 50 kDa MWCO was then selected for the other tests. Tests carried out with larger volumes in real conditions (up to 1500 L) made it possible to obtain a hydrocarbon concentration in the permeate of less than 0.1 mg L^−1^ for FCV = 50, and a stable permeability of 100 L h^−1^ m^2^ bar^−1^. In all these cases, as expected, there was a large amount of suspended matter retention with a permeate turbidity lower than 1 NTU. In the distinct context of high-temperature condensate treatment, it appeared that both on a laboratory scale and with on-site tests, ultrafiltration resulted in a very high reduction in turbidity and hydrocarbon concentration in the permeate. TMP and MWCO were the key parameters where a value of 1 bar and 50 kDa (52 channels), respectively, produced the best results for this process. The regeneration of the membranes was effective with conventional chemical cleaning (acid–base) and allowed the dimensioning of the industrial unit to be installed on site. From the results obtained and the parameters defined, MWCO, TMP, and membrane geometry, a first approximation of the industrial membrane area to treat 300 m^3^ h^−1^ was carried out at around 500 m^2^ for ultrafiltration at constant permeate flux. This value, as well as the associated cost, remain to be validated by further tests, in order to consider the variability of the effluent over several months which the authors hope to carry out in the near future.

## Figures and Tables

**Table 1:** Composition of effluents.

Effluent	Chronicle Pollution	Accidental Pollution	Petrochemical Plant
Hydrocarbon (mg L^−1^)	0.2	>50	0.2
Turbidity (NTU)	0.66	1.13	2.12
Conductivity (µS cm^−1^)	96.4	12.5	4.41
pH	5.95	6.98	6.3

**Table 2:** Characteristics of the membranes.

Molecular Weight Cut Off	150 kDa	50 kDa	50 kDa
Number of channels	19	7	52
Surface (m^2^)	0.24	0.15	0.42
Channel diameter (mm)	3.5	6	2.2
Length (m)	1178	1178	1178

**Table 3:** Analysis of the samples [Chronicle pollution, 150 kDa].

Volume Concentration Factor	Sample	pH	Conductivity (µS cm^−1^)	Turbidity (NTU)	[HC] (mg L^−1^)
1	Concentrate	5.95	96.4	0.66	0.2
	Permeate	6.65	24.3	0.21	0.4
2	Concentrate	6.5	42.1	1.73	0.8
	Permeate	5.95	88.5	0.14	0.3
70	Concentrate	6.3	96.4	26.7	15
	Permeate	5.82	52.3	0.34	0.2

**Table 4:** Analysis of the samples [Chronicle pollution mixed with gasoline or fuel].

Experiment	Transmembrane Pressure (bar)	pH	Conductivity (µS cm^−1^)	Turbidity Retention (%)		Permeate [HC] (mg L^−1^)	
				Initial	Final	Initial	Final
Chronic pollution + gasoline (100 ppm), 150 kDa	1	6.3	54.1	52	97.5	<0.1	<0.1
Chronic pollution + gasoline (100 ppm), 150 kDa	1.5	6.2	41.8	87.5	97.9	<0.1	<0.1
Chronic pollution + fuel (100 ppm), 150 kDa	1.5	6.2	28.5	68.3	99.4	<0.1	<0.1
Chronic pollution + gasoline (100 ppm), 50 kDa (7 channels)	1.5	6.8	35.9	93.8	99.8	<0.1	<0.1

**Table 5:** Analysis of the samples [Accidental pollution].

Experiment	Transmembrane Pressure (bar)	pH	Conductivity (µS cm^−1^)	Turbidity Retention (%)		Permeate [HC] (mg L^−1^)	
				Initial	Final	Initial	Final
Accidental pollution 50 kDa (7 channels)	1	6.7	55.2	31.3	97.4	<0.1	<0.1
Accidental pollution 50 kDa (52 channels)	1	6.9	12.5	79.6	90.6	<0.1	<0.1

**Table 6:** Analysis of the samples [Petrochemical plant].

Initial Volume (L)	Transmembrane Pressure (bar)	pH	Conductivity (µS cm^−1^)	Turbidity Retention (%)		Permeate [HC] (mg L^−1^)	
				Initial	Final	Initial VCF	Final VCF
400	2	6.7	4.4	45.6	96.1	<0.1	<0.1
1500	2	6.8	6.6	81	92.8	<0.1	<0.1

## Data Availability

The data presented in this study are available on request from the corresponding author.
